# Fast-CenLaneNet: A Lightweight Instance Segmentation-Based Network for Real-Time Lane Detection

**DOI:** 10.3390/jimaging12070320

**Published:** 2026-07-13

**Authors:** Qidong Han, Shuo Feng, Yang Gao, Mengyao Li, Teng Meng, Ke Li, Yuhao Yang

**Affiliations:** 1 School of Automobile, Chang’an University, Xi’an 710064, China; qd_han2020@163.com (Q.H.); nchygy@chd.edu.cn (Y.G.); 2 School of Construction Machinery, Chang’an University, Xi’an 710064, China; limengyaomail@163.com (M.L.); like-chd@chd.edu.cn (K.L.); yangyuhao_chd@163.com (Y.Y.); 3 School of Modern Post, Xi’an University of Posts and Telecommunications, Xi’an 710061, China; mengteng@aliyun.com

**Keywords:** lane detection, lightweight network, instance segmentation, attention mechanism

## Abstract

Lane detection is a critical component of autonomous driving systems, requiring both high accuracy and real-time performance under complex driving scenarios. Unlike current methods that rely on predefined lane counts, instance segmentation methods can handle an arbitrary number of lanes, making them more adaptable in real-world applications. However, this flexibility typically relies on dense pixel-level predictions, which necessitate large-scale networks and result in prohibitively high computational costs, hindering deployment on embedded platforms. To address these challenges, we present Fast-CenLaneNet, a lightweight architecture that improves inference efficiency while maintaining detection accuracy. Specifically, we design a lightweight backbone to reduce model parameters and computational cost, propose a learnable spatial similarity attention module to capture spatial dependencies within lane regions and enhance feature discriminability, and construct multi-branch output heads with Ghost convolutions to refine lane-related features with low computational overhead. Experiments on the TuSimple and CULane benchmarks demonstrate that Fast-CenLaneNet achieves a favorable accuracy–efficiency trade-off. On TuSimple, Fast-CenLaneNet obtains 96.40 ± 0.06% accuracy and 162.7 ± 6.8 FPS with 4.7 M parameters and 9.9 GFLOPs. Compared with CenLaneNet, it reduces the number of parameters by 89.1% and improves forward inference speed by 107.5%, with an accuracy decrease of only 0.08 percentage points.

## 1. Introduction

Autonomous driving technology is fundamentally reshaping the transportation landscape. Its performance critically depends on the vehicle’s capacity to perceive the surrounding environment [1,2]. As a foundational module, lane detection must ensure high accuracy, robustness, and real-time performance under diverse and challenging conditions, such as drastic illumination changes, severe occlusions, ambiguous road markings, and dynamic lane-changing maneuvers [3].

Lane detection algorithms are generally categorized into two categories: traditional and deep learning-based methods. Traditional methods typically rely on hand-crafted low-level features, including color spaces [4] and edge information [5], which are then processed through mathematical transformations, like the Hough transform [6,7], followed by curve fitting for lane modeling [8,9,10,11]. However, these methods often lack robustness and accuracy in complex scenarios.

Deep learning-based lane detection has achieved significant advances in recent years, owing to its powerful feature representation capabilities. These methods can be broadly classified into several categories: detection-based [12,13,14], parametric curve-based [15], and segmentation-based methods [16,17,18]. Despite substantial progress, critical challenges remain, particularly in lane instance discrimination [19]. Although some algorithms detect lane points before grouping them into instances [20,21], accurately assigning points to their respective lanes remains a difficult problem.

To solve this problem, a common solution is to treat lane detection as a multi-class detection problem [16,22], where lanes are assigned to a fixed number of classes. However, this approach is limited to a predefined number of lanes and is susceptible to class ambiguity caused by varying spatial relationships between the vehicle and the lanes [23]. Although anchor-based methods can alleviate this issue, they often require heuristic anchor designs or complex post-processing [24,25].

An alternative strategy is to treat lane detection as an instance segmentation task [26,27], which enables the identification of an arbitrary number of lane instances without predefined categories. However, these methods typically rely on large-scale networks and computationally intensive post-processing, hindering their deployment on resource-constrained devices. To improve efficiency, an anchor-free instance segmentation method based on lane center point estimation was proposed [23], which reduces lane ambiguity arising from variations in spatial relationships.

Nevertheless, the trade-off between accuracy and efficiency remains unresolved for instance segmentation-based lane detection. This paradigm relies on dense pixel-level representations and discriminative embeddings to separate adjacent slender lanes, which often requires high-capacity backbones and increases computational cost. Directly compressing the backbone or prediction heads can improve efficiency but may degrade embedding quality, weaken spatial continuity, and increase confusion between neighboring lane instances. Common attention modules also have limitations in this setting: dense spatial attention introduces considerable memory and computational overhead, whereas channel attention and direct similarity-based reweighting may be insufficient for modeling discriminative spatial dependencies in slender lane structures. Therefore, preserving lane-instance discrimination while reducing model complexity remains a key challenge for instance segmentation-based lane detection.

To address this challenge, this study proposes Fast-CenLaneNet, a lightweight instance segmentation-based lane detection method built upon the CenLaneNet framework [23]. Fast-CenLaneNet preserves the strengths of instance segmentation while significantly reducing the number of parameters and computational complexity. A lightweight backbone network is incorporated, and a learnable spatial similarity attention (LSSA) module is introduced to enhance discriminative feature representation. Experiments on public benchmark datasets demonstrate that Fast-CenLaneNet achieves competitive accuracy while substantially improving inference speed.

The main contributions of this study are summarized as follows:

(1) It develops a lightweight backbone network by integrating efficient operators, substantially reducing the number of parameters and computational complexity while maintaining feature representation capability.

(2) It proposes an LSSA that effectively captures spatial dependencies and improves feature discriminability for lane structures.

(3) It designs a lightweight instance segmentation-based lane detection method and conducts extensive experiments on public benchmark datasets. The experimental results indicate that Fast-CenLaneNet achieves a favorable balance between accuracy and efficiency.

This paper is organized as follows. Section 2 reviews related work on lane detection. Section 3 describes the proposed algorithm in detail. Section 4 presents the experimental setup and results. Finally, Section 5 concludes the paper.

## 2. Related Works

### 2.1. Traditional Methods

Traditional lane detection methods rely on hand-crafted low-level features and generally follow a two-stage pipeline: feature extraction and curve fitting [28]. In the first stage, image filters such as Sobel [29], Canny [5,30], or steerable filters [31,32,33] are commonly applied to identify lane features. The second stage involves geometric modeling to reconstruct the lanes from the extracted features. For example, Niu et al. [7] employed the Hough transform combined with clustering to fit lane curves. Mammeri et al. [6] integrated progressive probabilistic Hough transform with maximally stable extremal region to enhance detection accuracy. Parametric curve models have also been widely used for structured lane representation. Li et al. [8] adopted cubic B-spline curves for accurate lane modeling, whereas Wang et al. [9] proposed a Catmull–Rom spline-based model capable of flexibly generating lanes of arbitrary shapes using control points. Despite these advances, traditional methods generally perform poorly under complex road conditions.

### 2.2. Deep Learning Methods

Deep learning-based methods have achieved remarkable success in handling complex driving scenarios. These methods are generally categorized as anchor-based [24,25,34] or anchor-free on design principles, and as detection-based [12,13,14], parametric curve-based [15], or segmentation-based [16,17,18] on technical implementation.

Anchor-based algorithms often rely on dense predefined anchors and involve complex post-processing, leading to high computational costs. Anchor-free methods avoid the need for predefined anchors. Parametric curve-based methods [15,35] model lanes as parameterized curves, with networks directly predicting curve parameters. However, the parametric form imposes strong constraints, limiting its flexibility in handling lanes with complex topologies. Detection-based methods [12,13] locate lane keypoints, whereas segmentation-based methods [16,17] classify each pixel as lane or background. Both approaches often treat lane detection as a fixed-class classification problem to facilitate the assignment of predicted points to lane instances. Consequently, they are limited to detecting a preset number of lanes and are prone to class ambiguity when the relative spatial relationship between the vehicle and lanes varies [23].

Recent Transformer-based lane detectors, such as Laneformer and LDTR, enhance global lane modeling through row–column attention or structured lane representations [36,37]. However, these methods may introduce complex attention operations, making them less suitable for lightweight real-time lane detection. Diffusion-based lane detection has also been explored. For example, DiffusionLane formulates lane detection as a denoising diffusion process in the lane parameter space [38]. However, this direction remains at an early stage and is not primarily designed for lightweight real-time inference.

Instance segmentation methods [26,27] differentiate lane instances by assigning an embedding vector to each pixel, thus eliminating the need for a predefined number of lanes. While effective, these methods typically rely on large-scale network models and incur substantial computational overhead. Although recent work [23] has sought to enhance efficiency through center point-based strategies, the overall network design remains relatively heavy. This study proposes a lightweight anchor-free instance segmentation method for lane detection, aimed at reducing the inherent computational complexity of such approaches. To provide a concise overview of the reviewed methods, Table 1 summarizes representative lane detection paradigms and their main characteristics and limitations.

## 3. Methodology

**Figure 1:**
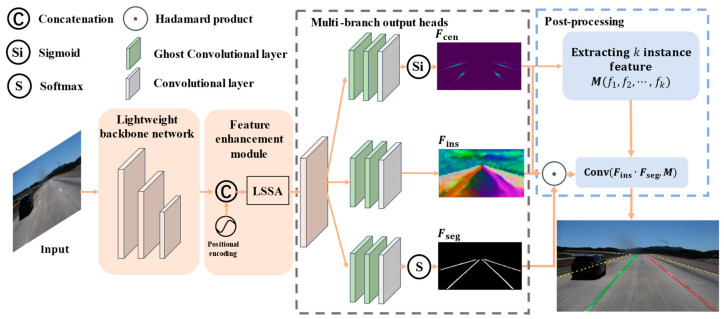
Overview of Fast-CenLaneNet.

Figure 1 illustrates the overall architecture of Fast-CenLaneNet, a fast center-based lane instance segmentation network built upon the CenLaneNet framework [23]. Centered on lightweight feature extraction, the architecture integrates four key components: a lightweight backbone for efficient feature extraction, a feature enhancement module for strengthening lane-related representations, multi-branch output heads for lane prediction, and a post-processing module for lane instance reconstruction.

### 3.1. Lightweight Backbone Network

To balance efficiency and feature extraction capabilities, we design a lightweight backbone network (LBN) based on the residual block [39]. By integrating Ghost convolution [40] and Self-Batch normalization [41], the backbone reduces computational complexity while preserving informative lane-related features.

**Figure 2:**
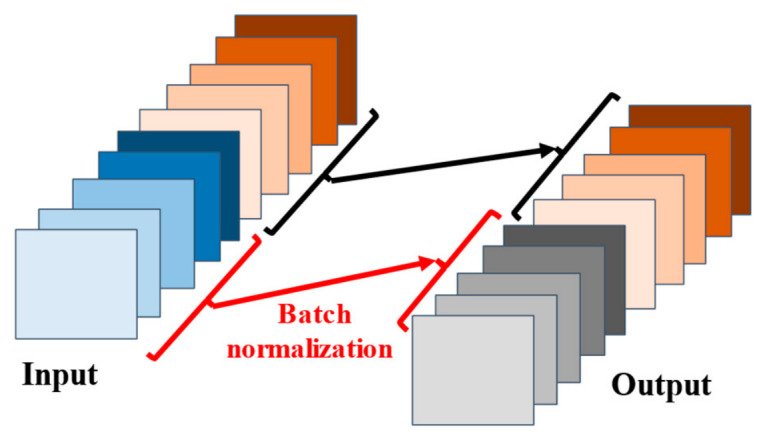
Self-BN module.

The Self-BN module [41], shown in Figure 2, is used at the input stage to preserve local details during feature processing. Given the input feature $X_{1}\in R^{B\times C\times H\times W}$, the module splits it along the channel dimension into two sub-features, $X_{a}$ and $X_{b}$, where $X_{a},\;X_{b}\in R^{B\times \frac{C}{2}\times H\times W}$. Applying batch normalization only to $X_{a}$ while directly preserving $X_{b}$, the module produces the output feature $Y_{1}\in R^{B\times C\times H\times W}$ by concatenating the two branches along the channel dimension.

**Figure 3:**
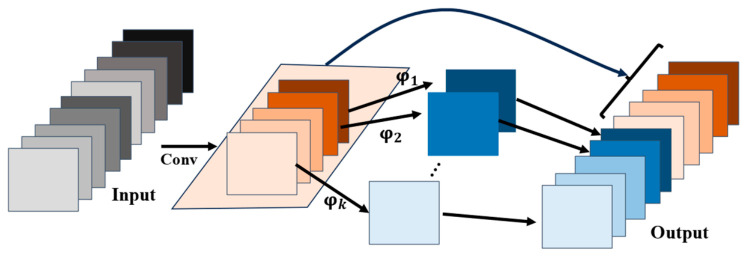
Ghost convolution.

To further reduce computational cost, Ghost convolution [40] is embedded into the residual blocks. As shown in Figure 3, let $X_{2}\in R^{C_{in}\times H\times W}$ and $Y_{2}\in R^{C_{out}\times H_{1}\times W_{1}}$ denote the input and output feature maps, respectively. Ghost convolution generates the output features in two stages. In the first stage, a primary convolution produces the intrinsic features $F_{p}$:

(1) $$F_{p}=\sigma \left(BN\left(Conv\left(X_{2}\right)\right)\right),\;F_{p}\in R^{\frac{C_{out}}{s}\times H_{1}\times W_{1}}$$

where $Conv\left(\cdot \right)$, $BN\left(\cdot \right)$, and $\sigma \left(\cdot \right)$ represent the convolution operation, batch normalization, and activation function, respectively. In this study, $\sigma \left(\cdot \right)$ is set to ReLU, and the scale factor s is set to 2.

In the second stage, a cheap depthwise convolution ($DWConv\left(\cdot \right)$) generates redundant features $F_{c}\in R^{C_{c}\times H_{1}\times W_{1}}$, where ${C_{c}=C}_{out}-C_{out}/s$:

(2) $$F_{c}=\sigma \left(BN\left(DWConv\left(F_{p}\right)\right)\right)$$

Concatenating the intrinsic features $F_{p}$ and redundant features $F_{c}$ along the channel dimension yields the final output $Y_{2}$:

(3) $$Y_{2}=Concat\left(F_{p},F_{c}\right)$$

Here, $Concat\left(\cdot \right)$ denotes channel-wise concatenation.

For clarity, Ghost convolution with convolution, BN, and ReLU in both stages is denoted as $GhostConvR\left(\cdot \right)$. Replacing $\sigma \left(\cdot \right)$ in both stages with identity mapping while retaining $BN\left(\cdot \right)$ gives $GhostConvI\left(\cdot \right)$. Removing both $\sigma \left(\cdot \right)$ and $BN\left(\cdot \right)$ from both stages gives $GhostConv\left(\cdot \right)$.

**Figure 4:**
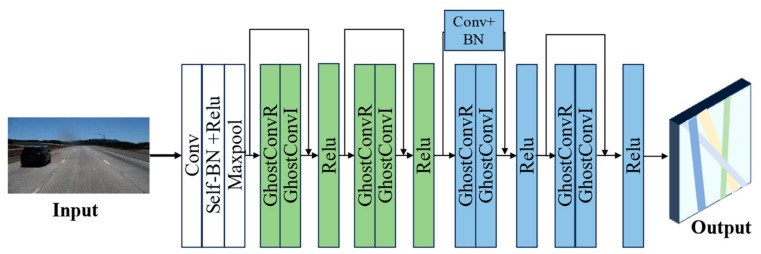
Architecture of the lightweight backbone network.

Given that lane detection mainly depends on relatively low-level semantic and structural features, the proposed LBN adopts two lightweight residual stages, highlighted by the green and blue regions in Figure 4. The detailed configuration of the LBN is summarized in Table 2. This compact design reduces both the number of parameters and computational cost while effectively capturing lane-related semantic features.

### 3.2. Feature Enhancement Module

**Figure 5:**
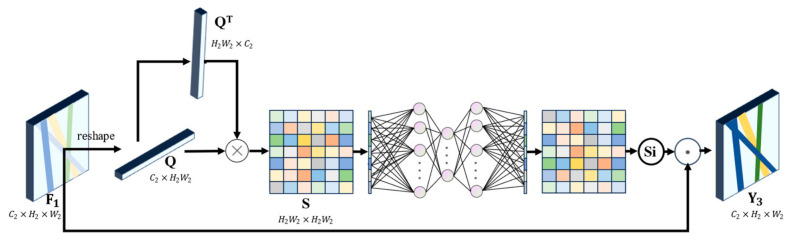
Learnable spatial similarity attention.

Improving long-range perception for slender structures often increases network complexity [42]. To enhance lane-related features more efficiently, we introduce a feature enhancement module whose core component is the LSSA. While the position attention module (PAM) [43] directly uses similarity scores for feature reweighting, LSSA introduces fully connected layers to selectively emphasize discriminative features, which is crucial for slender lane structures. This selection mechanism enables the model to focus on lane-relevant spatial dependencies while suppressing less informative background responses. The overall structure of LSSA is illustrated in Figure 5.

Specifically, let the output feature map from the LBN be denoted as $X_{3}\in R^{C_{1}\times H_{2}\times W_{2}}$, where $C_{1}=128$ and $H_{2}\times W_{2}=32\times 64$. Before LSSA, a fixed two-dimensional sinusoidal positional encoding [44] is concatenated with $X_{3}$ along the channel dimension:

(4) $$F_{1}=Concat\left(X_{3},P\right)$$

where $P\in R^{C_{1}\times H_{2}\times W_{2}}$, $F_{1}\in R^{C_{2}\times H_{2}\times W_{2}}$, and $C_{2}=256$. The positional encoding is fixed and contains no learnable parameters.

Within the LSSA, $F_{1}$ is reshaped into $Q\in R^{C_{2}\times \left(H_{2}W_{2}\right)}$, and the spatial similarity matrix S is computed as:

(5) $$S=\frac{Q^{T}Q}{C_{2}^{m}},S\in R^{{(H}_{2}W_{2})\times {(H}_{2}W_{2})}$$

Here, $Q^{T}Q$ denotes the pairwise spatial similarity matrix computed by matrix multiplication, and $C_{2}^{m}$ is used to scale the similarity response, where m is set to 0.5 according to the sensitivity analysis in Section 4.5.

Subsequently, the spatial similarity matrix S is passed through a series of fully connected layers:

(6) $$R=Relu\left(W_{1}\left(S\right)\right),\;R\in R^{{(H}_{2}W_{2})}$$

(7) $$F_{2}=\mathrm{Sigmoid}\left(Re\left(W_{3}\left(Relu\left(W_{2}\left(R\right)\right)\right)\right)\right)$$

where $W_{1}\left(\cdot \right)$ is a row-wise fully connected layer that compresses each $H_{2}W_{2}$-dimensional similarity vector into a scalar. $W_{2}\left(\cdot \right)$ and $W_{3}\left(\cdot \right)$ form a two-layer bottleneck mapping from $H_{2}W_{2}$ to ${(H}_{2}W_{2})/2$ and then back to $H_{2}W_{2}$. $Re\left(\cdot \right)$ represents the reshaping operation from $R^{\left(H_{2}W_{2}\right)}$ to $R^{H_{2}\times W_{2}}$. $Relu\left(\cdot \right)$ and $\mathrm{Sigmoid}\left(\cdot \right)$ are activation functions.

Multiplying the resulting attention map $F_{2}$ element-wise with the original feature map $F_{1}$ enhances the spatial feature representation:

(8) $$Y_{3}=F_{1}⨀F_{2},\;\;Y_{3}\in R^{C_{2}\times H_{2}\times W_{2}}$$

LSSA is designed to enhance feature discriminability for instance segmentation-based lane detection, where accurate lane separation depends on distinguishing pixel-level features from adjacent lanes and background regions. The similarity matrix S measures pairwise affinity among spatial features, providing cues for identifying lane-consistent regions and separating adjacent lanes from background responses. Nevertheless, raw pairwise similarities are not necessarily discriminative for lane instance separation because high affinity may also arise between adjacent lane markings, homogeneous background regions, or non-lane objects with similar appearances. The FC mapping, therefore, acts as a learnable selection step to refine these similarity relationships and emphasize affinities that are more beneficial to lane separation. Unlike standard self-attention, which aggregates value features from all spatial positions, LSSA uses the refined similarity information to generate a spatial refinement map, thereby enhancing lane-discriminative dependencies with lower structural complexity. Quantitative improvements over other attention modules are reported in the experimental section.

The computational cost of LSSA is mainly determined by the spatial similarity matrix S. For the input resolution of 256 × 512, the spatial resolution of $F_{1}$ is $H_{2}\times W_{2}=32\times 64$, and thus ${N=H}_{2}W_{2}=2048$. Therefore, $S\in R^{N\times N}$ has a size of 2048 × 2048, containing approximately 4.19 M intermediate elements and requiring about 16.0 MiB of activation memory for storing S in FP32 precision. The dominant computation comes from $Q^{T}Q$. With $C_{2}=256$, this operation requires approximately 2.15 GFLOPs. Including the subsequent FC mapping, the overall forward computational cost of LSSA is approximately 2.16 GFLOPs per image. Although LSSA has quadratic complexity with respect to N, applying it at the 32 × 64 feature map resolution keeps the additional cost bounded in the proposed lightweight lane detection framework.

### 3.3. Output Heads and Post-Processing

Following [23], the enhanced feature map $Y_{3}$ is further processed by a multi-branch prediction head together with the corresponding post-processing strategy to generate the final lane instances.

Specifically, the enhanced feature map $Y_{3}$ is first refined as:

(9) $$F_{3}=Conv\left(GhostConvR\left(Y_{3}\right)\right)$$

where $GhostConvR\left(\cdot \right)$ denotes a Ghost convolution in which both stages retain BN and ReLU activation.

After being upsampled by the bilinear upsampling module ($BUM\left(\cdot \right)$), the refined feature $F_{3}$ is processed in parallel by three task-specific branches to produce the lane center heatmap $F_{cen}\in R^{1\times H\times W}$, the semantic segmentation map $F_{seg}\in R^{C\times H\times W}$, and the instance embedding map $F_{ins}\in R^{D\times H\times W}$, where D=6 follows the setting of CenLaneNet [23].

The formulation for each branch is as follows:

Lane center heatmap:

(10) $$F_{cen}=\mathrm{Sigmoid}\left({Conv}_{1}\left({GhostConv}_{2}\left({GhostConv}_{1}\left(\mathrm{BUM}\left(F_{3}\right)\right)\right)\right)\right)$$

Semantic segmentation map:

(11) $$F_{seg}=\underset{C}{Softmax}\left({Conv}_{2}\left({GhostConv}_{4}\left({GhostConv}_{3}\left(\mathrm{BUM}\left(F_{3}\right)\right)\right)\right)\right)$$

Instance embedding map:

(12) $$F_{ins}={Conv}_{3}\left({GhostConv}_{6}\left({GhostConv}_{5}\left(\mathrm{BUM}\left(F_{3}\right)\right)\right)\right)$$

Based on the three prediction branches, the post-processing stage further decodes the final lane instances [23]. Specifically, the instance embedding map is $L_{2}$-normalized along the channel dimension:

(13) $${\tilde{F}}_{ins}\left(p\right)=\frac{F_{ins}\left(p\right)}{{\left\|F_{ins}\left(p\right)\right\|}_{2}+\epsilon }$$

where p denotes a pixel location and $\epsilon =10^{-6}$ is used for numerical stability.

Candidate lane centers are extracted from $F_{cen}$ using max pooling-based non-maximum suppression:

(14) $${\tilde{F}}_{cen}={NMS}_{k}\left(F_{cen}\right)$$

where ${NMS}_{k}\left(\cdot \right)$ is implemented by a k×k max pooling operation with stride 1 and padding $\left\lfloor k/2\right\rfloor$, where $\left\lfloor \cdot \right\rfloor$ denotes the floor operation. No additional absolute heatmap threshold is used. Instead, the top $K_{0}$ responses after NMS are selected as candidate centers. In our experiments, k=11 and $K_{0}=20$.

For each candidate center $q_{j}=\left(x_{j},y_{j}\right)$, its embedding vector is sampled from the normalized instance embedding map:

(15) $$e_{j}={\tilde{F}}_{ins}\left(q_{j}\right)$$

To remove duplicate candidate centers from the same lane, pairwise cosine similarities are computed by the inner product between normalized center embeddings. Candidates with similarity higher than $\tau_{c}=0.5$ are regarded as belonging to the same lane center group, and only the candidate with the highest center response in each group is retained. The remaining K center embeddings are used as lane prototypes:

(16) $$M=\left[m_{1},m_{2},\ldots ,m_{K}\right]\in R^{D\times K}$$

Then, the prototype matching score map is computed by a 1 × 1 convolution whose weights are given by the transposed prototype matrix $M^{T}$:

(17) $$S=Conv\left({\tilde{F}}_{ins};M^{T}\right),S\in R^{K\times H\times W}$$

Here, the convolution uses kernel size 1×1, stride 1, padding 0, and no bias. Equivalently, for each pixel p,

(18) $$S_{i}\left(p\right)=m_{i}^{T}{\tilde{F}}_{ins}\left(p\right),i=1,\cdots ,K.$$

The preliminary instance assignment is obtained by selecting the prototype with the maximum similarity:

(19) $$L\left(p\right)=arg{max}_{i\in \left\{1,\cdots ,K\right\}}S_{i}\left(p\right)$$

Here, the argmax is taken over the K lane prototypes. In $F_{seg}$, channel 0 denotes background and channel 1 denotes lane foreground. The final mask for the i-th lane instance is

(20) $$I_{i}\left(p\right)=I\left[L\left(p\right)=i\right]\cdot I\left[arg{max}_{c\in \left\{0,1\right\}}F_{seg}^{c}\left(p\right)=1\right]\cdot I\left[{{max}_{l\in \left\{1,\cdots ,K\right\}}S}_{l}\left(p\right)>\tau_{s}\right]$$

where $I\left[\cdot \right]$ denotes the indicator function, which equals 1 if the condition inside the brackets is true and 0 otherwise. The first term assigns pixels to the i-th prototype, the second term enforces the semantic foreground constraint, and the third term removes pixels whose maximum prototype similarity is lower than $\tau_{s}$. In our experiments, $\tau_{s}=0.8$ is the pixel-level prototype similarity threshold.

For TuSimple evaluation, each decoded instance mask is resized to the original image resolution and converted into lane coordinates at the predefined vertical coordinates specified by the evaluation protocol. For each sampled row, the horizontal coordinate is obtained by averaging the x-coordinates of pixels belonging to the corresponding instance. If no valid pixel exists at a sampled row, linear interpolation or first-order polynomial extrapolation is used when neighboring valid points are available; otherwise, the value is set to −2, following the TuSimple evaluation protocol. For CULane evaluation, each instance mask is converted into an ordered polyline by scanning the mask from bottom to top at a fixed row interval and computing the mean horizontal coordinate of the lane pixels at each selected row. The resulting $\left(x,y\right)$ points are then rescaled to the original image resolution for evaluation.

### 3.4. Loss Function

The overall training objective is formulated by combining the three supervision terms:

(21) $$L=\alpha_{0}L_{cen}+\alpha_{1}L_{seg}+\alpha_{2}L_{ins}$$

(22) $$L_{seg}=\beta_{1}L_{dice}+\beta_{2}L_{ce}$$

where the hyperparameters $\alpha_{0}$, $\alpha_{1}$, $\alpha_{2}$, $\beta_{1}$, and $\beta_{2}$ are used to balance the contributions of different supervision terms. Specifically, $\alpha_{0}$, $\alpha_{1}$, and $\alpha_{2}$ are set to 50, 0.05, and 0.0001, respectively, and $\beta_{1}=\beta_{2}=0.5$. $L_{cen}$ is implemented using the lane center heatmap loss [23,45], $L_{seg}$ is defined as the combination of Dice loss [46] and cross-entropy loss, and $L_{ins}$ is implemented as the Large Margin Cosine Loss [23,47].

## 4. Experiments

### 4.1. Datasets

This study evaluates the proposed method on two widely used benchmark datasets. The first is the TuSimple benchmark [48], which contains 6408 highway images at a resolution of 720 × 1280 pixels. The dataset is split into 3626 training and 2782 test images. The second is the more challenging CULane benchmark [16], comprising 132,235 images at 1640 × 590 pixels. It covers diverse driving environments, including urban streets, rural roads, and highways, and is divided into 88,880 training, 9675 validation, and 34,680 test images.

### 4.2. Implementation Details

Models were trained from scratch on both the TuSimple and CULane datasets without using pretrained weights. The Adam optimizer was adopted with β_1_ = 0.9 and β_2_ = 0.999. The learning rate was scheduled using the OneCycle policy [49]. Specifically, the learning rate was initialized at 5 × 10^−5^, increased to 1 × 10^−3^ during the first 10% of the training iterations, and then decreased to 5 × 10^−9^ using cosine annealing. The momentum coefficient was scheduled in the opposite direction, varying between 0.95 and 0.85 during training.

All images from both TuSimple and CULane were directly resized to 256 × 512 before being fed into the network, without padding or aspect ratio-preserving cropping. During training, data augmentation was implemented using imgaug. The detailed augmentation settings are listed in Table 3.

Training was conducted for 100 epochs, with a batch size of 5 for TuSimple and 24 for CULane. On the training platform listed in Table 4, training Fast-CenLaneNet took approximately 2 h on TuSimple and 46 h on CULane. The implementation was based on PyTorch 1.11.0, CUDA 11.3, and cuDNN 8.2.0. For reproducibility, all repeated experiments were conducted with the same five fixed random seeds, and a control variable protocol was adopted in the ablation and sensitivity studies, where only the evaluated module or hyperparameter was changed. Across the repeated runs, the training process remained stable, and the metrics showed only minor fluctuations in the later epochs. For inference time measurement, all models were evaluated on the inference platform listed in Table 4 with a batch size of 1 under FP32 precision, and no deployment-specific acceleration was used. The results of repeated experiments are reported as mean ± standard deviation.

### 4.3. Evaluation Metrics

For the TuSimple dataset, the official evaluation protocol is adopted; the metrics of accuracy (Acc), false positive rate (FPR), and false negative rate (FNR) are reported [48]. Specifically, the evaluation is conducted on predefined vertical coordinates by comparing the predicted lane points with the corresponding ground-truth points under a fixed distance threshold ϵ. The Acc is defined as the average proportion of predicted points whose Euclidean distance to the corresponding ground-truth points is within the threshold. A ground-truth lane is regarded as correctly detected when the matching accuracy exceeds the official criterion (0.85); otherwise, it is counted as a false negative, while unmatched predicted lanes are counted as false positives. Accordingly, FNR measures the proportion of missed ground-truth lanes, and FPR measures the proportion of unmatched predicted lanes.

For the CULane dataset, the official F-measure is adopted for evaluation [16]. True positives (TPs), false positives (FPs), and false negatives (FNs) are determined according to the intersection over union (IoU) between predicted lanes and ground-truth lanes, where the IoU threshold is set to 0.5. Precision and recall are computed based on TPs, FPs, and FNs, and the F-measure is obtained as their harmonic mean.

### 4.4. Comparison with State-of-the-Art Methods

To evaluate the performance of the proposed method, we compare it with state-of-the-art methods on the TuSimple and CULane datasets. The detection performance metrics of baseline methods, including Acc, FPR, FNR, and F-measure, are taken from the original papers. For a fair efficiency comparison, values marked with * in tables were re-measured by us, whereas unmarked values were taken from the original papers. Specifically, starred FPS values were re-measured on the inference platform described in Section 4.2, and starred Params and FLOPs values were computed using torchprofile with the official input resolution of each method. Each multiply–accumulate operation is counted as two floating-point operations.

Table 5 reports the comparison results on the TuSimple dataset, including both detection accuracy metrics and efficiency-related metrics. Specifically, Acc, FPR, and FNR are used to evaluate detection performance, while FPS, the number of parameters, and FLOPs are used to evaluate inference speed and computational complexity. We note that prior studies exhibit significant inconsistencies in efficiency evaluation: some measure the full pipeline including post-processing, whereas others only consider forward inference time. Moreover, some methods report inference time using an all-ones matrix as input, which tends to overestimate practical speed. Considering these factors, forward inference time more directly reflects the intrinsic computational efficiency of a model, whereas post-processing time is highly dependent on hardware, software implementation, and data structures, and, therefore, should not be directly compared without a unified implementation. To provide a fair and realistic evaluation, we adopt the following protocol on TuSimple: after a 10-frame warm-up, each model evaluated in this work is tested on 100 consecutive real images, and the average forward inference time is recorded and converted to FPS.

As shown in Table 5, Fast-CenLaneNet achieves 96.40% accuracy, with an FPR of 0.043 and an FNR of 0.029. Its accuracy is only 0.13% lower than that of SCNN [16] and TSA-LNet [3], which achieve the highest accuracy of 96.53%. Meanwhile, Fast-CenLaneNet achieves the highest forward inference speed among the compared methods, reaching 162.7 FPS with only 4.7 M parameters and 9.9 GFLOPs. Compared with CenLaneNet [23], Fast-CenLaneNet increases the forward inference speed by 107.5% and reduces the number of parameters by 89.1%, while maintaining an accuracy drop of only 0.08%. Although no specific optimization is applied to the post-processing stage, the full pipeline including post-processing still achieves 114 FPS, satisfying practical real-time requirements. These results indicate that Fast-CenLaneNet improves inference speed while maintaining comparable detection accuracy, and the reduced computational workload may also benefit the performance–power trade-off in real-time lane detection.

As shown in Table 6, Fast-CenLaneNet achieves a total F-measure of 72.7 on the more challenging CULane dataset. Although this result is lower than those of CenLaneNet [23] and TSA-LNet [3], which obtain 74.8 and 74.1, respectively, it remains higher than those of UltraLD [12], SCNN [16], FLLDM [52], and Enet-SAD [22], which obtain 72.3, 71.6, 71.0, and 70.8, respectively. Considering that Fast-CenLaneNet substantially improves inference speed and reduces model complexity, this result indicates that the proposed method maintains reliable detection performance on complex road scenes while improving computational efficiency.

For specific scenarios, Fast-CenLaneNet maintains results close to stronger baselines in several challenging categories. In the Dazzle category, it achieves 65.2, approaching CenLaneNet [23] (66.0) and outperforming TSA-LNet [3] (63.5). In the No line category, it obtains 47.9, which is close to CenLaneNet [23] (48.6) and higher than TSA-LNet [3] (46.1). In the Curve category, Fast-CenLaneNet reaches 68.8, approaching UltraLD [12] (69.5) and exceeding both CenLaneNet [23] and TSA-LNet [3] (66.5). These results indicate that the proposed method can preserve relatively stable detection performance under illumination interference, missing lane markings, and curved lane structures, despite its lightweight design. However, Crossroad remains the main limitation, where Fast-CenLaneNet produces 2866 false positives, compared with 1359 for CenLaneNet [23] and 2013 for TSA-LNet [3]. We attribute the increased false positives to the lightweight feature representation, which, while efficient, can activate texture-rich road markings and lane-like structures at intersections. Nevertheless, its stable performance in other challenging categories partly offsets this limitation.

**Figure 6:**
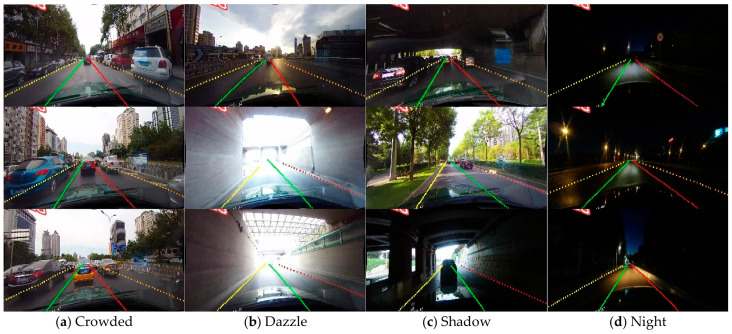
Qualitative results of Fast-CenLaneNet on representative CULane scenarios: (**a**) Crowded, (**b**) Dazzle, (**c**) Shadow, and (**d**) Night. Different colors indicate different predicted lane instances.

Representative qualitative results of Fast-CenLaneNet under challenging CULane scenarios are shown in Figure 6. The proposed method produces distinguishable lane instance predictions in crowded traffic, strong illumination interference, shadow regions, and nighttime scenes. These results qualitatively suggest that Fast-CenLaneNet can maintain lane instance discrimination under complex visual conditions.

Furthermore, as discussed in [23,27], traditional methods that rely on predefined lane counts are prone to class ambiguity in lane-change scenarios, posing potential risks for autonomous driving. In contrast, Fast-CenLaneNet adapts dynamically to variations in the lane number and spatial layout, thereby maintaining higher robustness under such challenging conditions. To quantitatively validate this capability, this study selects 285 lane-change frames across seven scenarios from the CULane dataset, comparing Fast-CenLaneNet with UltraLD [12] and Enet-SAD [22]. As shown in Table 7, Fast-CenLaneNet achieves more stable and consistent performance throughout the lane-change process, highlighting its practical value for real-world driving.

**Figure 7:**
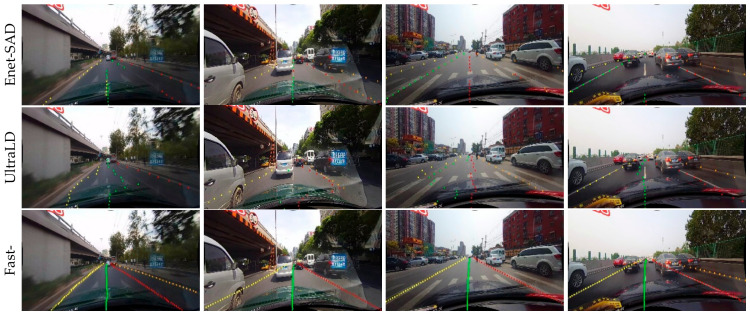
Qualitative comparison in lane-changing scenarios. Each row corresponds to one method: Enet-SAD, UltraLD, and Fast-CenLaneNet. Different colors indicate different predicted lane instances.

Figure 7 provides qualitative comparisons in lane-changing scenarios. During lane changes, the relative spatial relationship between adjacent lanes changes continuously, which can lead to lane instance confusion in baseline methods. For example, some predicted points may be incorrectly assigned to a neighboring lane instance. In contrast, Fast-CenLaneNet produces clearer instance separation and fewer visually observable lane instance assignment errors in these scenarios.

### 4.5. Module Analysis and Ablation Study

To evaluate the effectiveness of the proposed attention mechanism, this study compares LSSA with representative attention modules, including the Position Attention Module (PAM) [43], the Convolutional Block Attention Module (CBAM) [53], and Transformer-style multi-head self-attention (MHSA) [44]. PAM is used as a spatial attention baseline, CBAM is used as a channel–spatial hybrid attention baseline, and MHSA is used as a Transformer attention baseline. As shown in Table 8, LSSA achieves the best overall detection performance, with an accuracy of 96.40%, an FPR of 0.043, and an FNR of 0.029. Compared with PAM, LSSA obtains higher accuracy and lower FNR, indicating that directly using spatial similarity scores for feature reweighting is less effective than learnable similarity refinement for slender lane structures. Compared with CBAM, LSSA also achieves better lane detection performance, suggesting that sequential channel–spatial attention may be insufficient for modeling the pairwise spatial dependencies required for lane-instance separation. Although MHSA introduces global token-level attention through query key similarity computation and value aggregation, it does not explicitly refine lane-discriminative similarity relationships. In contrast, LSSA focuses on spatial similarity learning, which is more suitable for elongated and structured lane features.

The fully connected (FC) mapping structure in LSSA is further evaluated in Table 9. In this table, $N=H_{1}W_{1}$ denotes the number of spatial positions. Each notation in the FC mapping column represents the dimension sequence of the expansion mapping after row-wise similarity reduction, and each hyphen-connected transition corresponds to one FC layer. For example, N−N denotes a one-layer expansion mapping, whereas N−N/2−N denotes a two-layer bottleneck expansion mapping.

As shown in Table 9, the N−N/2−N mapping achieves the highest Acc of 96.40%, together with the lowest FPR of 0.043 and FNR of 0.029. Compared with the simpler N−N mapping, it slightly reduces FPS but improves Acc and reduces both FPR and FNR. When the mapping becomes deeper, as in N−N−N/2−N and N−N−N/2−N/2−N, the Acc does not improve further, and the FPS decreases. These results indicate that simply increasing the FC mapping depth does not necessarily enhance spatial similarity refinement. Therefore, the N−N/2−N mapping is selected as the expansion mapping in LSSA, as it achieves superior detection accuracy and error suppression while maintaining competitive inference efficiency.

The sensitivity of the scaling exponent m in LSSA is further evaluated in Table 10. As shown in Equation (5), m controls how strongly the pairwise similarity response is scaled. Among the tested settings, m = 0.5 achieves the highest accuracy and the lowest FPR and FNR, as shown in Table 10. Therefore, m = 0.5 is adopted as the default setting in LSSA.

Ablation experiments are conducted to evaluate the contributions of the proposed LBN and LSSA modules to Fast-CenLaneNet. As shown in Table 11, four configurations with or without LBN and LSSA are compared. For configurations without the LBN, the feature extraction module is replaced with the corresponding structure from ResNet-18 [39] using only its first two layers, while all other settings are kept unchanged. The experimental results show that the model incorporating both the LBN and LSSA achieves the best overall performance.

To further evaluate the statistical reliability of the ablation results, the statistical results are reported in Table 12. The confidence interval is computed for the mean accuracy difference between two compared configurations. A *p*-value lower than 0.05 is considered statistically significant.

Using the ResNet backbone alone as the baseline yields an accuracy of 96.13%, with 0.9 M parameters, 9.9 G FLOPs, and 189.9 FPS. Replacing it with the proposed LBN increases the accuracy to 96.34%. Meanwhile, the number of parameters is reduced from 0.9 M to 0.5 M, and the FLOPs decrease from 9.9 G to 7.7 G, corresponding to reductions of 44.4% and 22.2%, respectively. The paired *t*-test in Table 12 further shows that the accuracy improvement brought by LBN over ResNet is statistically significant, with a mean gain of 0.202 percentage points and a *p*-value of 0.002. These results demonstrate that LBN improves lane-related feature representation while reducing theoretical computational complexity. Although the FPS decreases from 189.9 to 164.6, this does not contradict the FLOP reduction, because practical inference latency is also affected by operator-level overhead.

The role of LSSA is further examined under different backbone settings. Integrating LSSA into the ResNet-based baseline improves the accuracy from 96.13% to 96.19%, with an increase of 0.06 percentage points. This improvement is accompanied by an increase in parameters from 0.9 M to 5.1 M and an increase in FLOPs from 9.9 G to 12.1 G, while the FPS decreases from 189.9 to 163.1. These results indicate that LSSA introduces additional computational cost, mainly due to spatial similarity modeling and FC mapping, while providing feature refinement for lane structures. When combined with the LBN backbone, LSSA also improves the Acc from 96.34% to 96.40%. However, as shown in Table 12, this improvement is not statistically significant under the five repeated runs, with a *p*-value of 0.103. This suggests that LBN already provides effective lane-related feature representations, leaving limited room for further accuracy improvement on the TuSimple benchmark.

This modest gain should also be interpreted together with the attention comparison in Table 8. Under the same LBN-based framework, PAM, CBAM, and MHSA obtain an accuracy of 96.29%, which is slightly lower than the LBN-only model with 96.34% accuracy. In contrast, LSSA increases the accuracy to 96.40% and achieves the lowest FNR of 0.029. These results suggest that, although the LBN backbone already has strong lightweight feature extraction capability, the proposed LSSA still provides complementary spatial refinement, whereas the other attention modules do not further improve accuracy in this lightweight framework. Therefore, the contribution of LSSA should be interpreted as complementary spatial feature refinement rather than a large standalone accuracy gain.

When LSSA is combined with LBN, the complete model achieves the best accuracy of 96.40%, with 4.7 M parameters, 9.9 G FLOPs, and 162.7 FPS. Compared with the LBN-only model, adding LSSA improves the accuracy by 0.06 percentage points, while increasing the FLOPs from 7.7 G to 9.9 G. Nevertheless, the FPS only changes slightly from 164.6 to 162.7, indicating that the additional latency introduced by LSSA remains limited in the lightweight framework. Compared with the ResNet + LSSA configuration, the complete model further improves the accuracy by 0.21 percentage points, while reducing parameters by 7.8% and FLOPs by 18.2%. The paired *t*-test also shows that LBN + LSSA significantly outperforms ResNet + LSSA, with a mean accuracy gain of 0.210 percentage points and a *p*-value lower than 0.001. These results indicate that LBN and LSSA are complementary: LBN mainly reduces model complexity, whereas LSSA enhances spatial feature discriminability. Their combination achieves a favorable balance between performance and inference speed.

## 5. Conclusions

This paper proposes Fast-CenLaneNet, a lightweight lane detection method designed to address the accuracy–efficiency trade-off in instance segmentation-based lane detection. The lightweight backbone and Ghost convolution-based multi-branch output heads reduce model complexity, while the LSSA module enhances spatial feature discrimination for slender lane structures. The experimental results show that Fast-CenLaneNet achieves 96.40% accuracy and 162.7 FPS on TuSimple with 4.7 M parameters and 9.9 GFLOPs, while obtaining a total F-measure of 72.7 on CULane. These results indicate that Fast-CenLaneNet improves inference efficiency while maintaining comparable detection accuracy. Future work will focus on reducing false positives in intersection-like scenes and optimizing the post-processing stage to further improve full-pipeline efficiency.

## Figures and Tables

**Table 1:** Summary of representative lane detection paradigms.

Category	Methods	Main Characteristics	Limitations
Traditional methods	[4,5,6,7,8,9,10,11]	Use hand-crafted features and geometric curve fitting.	Poor robustness in complex road scenes.
Detection-based methods	[12,13,14]	Detect lane keypoints or row-wise lane locations.	May require fixed lane assignments or additional grouping.
Parametric curve-based methods	[15,35]	Predict compact lane curve parameters.	Limited flexibility for complex lane topologies.
Segmentation-based methods	[16,17]	Perform pixel-level lane/background or lane class prediction.	Fixed-class design may cause class ambiguity.
Instance segmentation-based methods	[23,26,27]	Distinguish lane instances using embedding.	Dense representations and post-processing increase computational cost.
Transformer-based methods	[36,37]	Enhance global lane modeling with attention or structured representations.	Complex attention operations may affect lightweight real-time inference.
Diffusion-based methods	[38]	Formulate lane prediction as a denoising diffusion process.	Still at an early stage for lightweight real-time lane detection.

**Table 2:** Architecture of the LBN.

Stage	Main Branch	Shortcut	Fusion	Output Size (H × W × C)	Stride
Input	-	-	-	256 × 512 × 3	-
Stem	7 × 7 Conv + Self-BN + ReLU	-	-	128 × 256 × 64	2
Stem	3 × 3 MaxPool	-	-	64 × 128 × 64	2
LBN Stage 1	GhostConvR + GhostConvI	Identity	Add→ReLU	64 × 128 × 64	1
LBN Stage 1	GhostConvR + GhostConvI	Identity	Add→ReLU	64 × 128 × 64	1
LBN Stage 2	GhostConvR + GhostConvI	1 × 1 Conv + BN	Add→ReLU	32 × 64 × 128	2
LBN Stage 2	GhostConvR + GhostConvI	Identity	Add→ReLU	32 × 64 × 128	1
Output	LBN feature map $X_{3}$	-	-	32 × 64 × 128	-

**Table 3:** Data augmentation settings used during training.

Augmentation	Probability	Parameter Setting
Horizontal flipping	0.5	The image is horizontally flipped.
Channel shuffling	0.1	The RGB channel order is randomly shuffled.
Brightness adjustment	0.6	Multiplicative factor sampled from [0.85, 1.15]; additive offset sampled from [−10, 10].
Hue and saturation adjustment	0.7	Adjustment value sampled from [−10,10].
Blur	0.2	Motion blur or median blur randomly selected; kernel size sampled from 3 to 5.
Affine transformation	0.7	Translation sampled from [−10%,10%], rotation from [−10°,10°], and scaling from [0.8,1.2].

**Table 4:** Hardware configuration.

Item	Training	Inference
System	Red Hat^®^ Enterprise Linux 8.3	Microsoft^®^ Windows 11
GPU	NVIDIA^®^ A40	NVIDIA^®^ RTX3070Ti
CPU	Intel^®^ Xeon Platinum 8358	Intel^®^ Core i7-12700H
GPU memory	40 GB	8 GB

**Table 5:** Results on TuSimple.

Method	Acc (%)	FPR	FNR	Input Size (H × W)	FPS	Params (M)	FLOPs (G)
SCNN [16]	**96.53**	0.062	**0.018**	288 × 800	21.9 ± 1.7 *	20.7 *	218.4 *
FastDraw [50]	95.20	0.076	0.045	-	-	-	-
PolyLaneNet [51]	93.36	0.094	0.093	360 × 640	73.8 ± 5.6 *	**4.0 ** *	**3.** **6 ** *
CenLaneNet [23]	**96.48**	**0.031**	**0.025**	256 × 512	78.4 ± 2.3 *	43.1 *	12.2 *
FLLDM [52]	96.10	0.188	0.036	288 × 800	**150.5 ± 6.1 ** *	23.4	**2.8**
TSA-LNet [3]	**96.53**	**0.021**	**0.015**	256 × 512	**99.7 ± 2.1 ** *	**2.3**	35.6
Fast-CenLaneNet	**96.4** **0 ± 0.06**	**0.043 ** **± 0.003**	0.029 ± 0.001	256 × 512	**162.7 ± 6.8**	**4.7**	**9.9**

Note: The best, second, and third results are highlighted in bold black, bold red, and bold blue fonts, respectively. “-” indicates unavailable results. “*” indicates the corresponding values re-measured by us.

**Table 6:** Results on CULane. F-measure is reported for all categories except Crossroad, where false positives are reported.

Method	Total	Normal	Crowded	Dazzle	Shadow	No Line	Curve	Crossroad (FP↓)	Arrow	Night
SCNN [16]	71.6	90.6	69.7	58.5	66.9	43.4	64.4	**1990**	84.1	66.1
FastDraw [50]	-	85.9	63.6	57.0	59.9	40.6	65.2	7013	79.4	57.8
Enet-SAD [22]	70.8	90.1	68.8	60.2	65.9	41.6	65.7	**1998**	84.0	66.0
UltraLD [12]	72.3	90.7	70.2	59.5	69.3	44.4	**69.5**	2037	85.7	66.7
CenLaneNet [23]	**74.8**	**91.0**	**73.7**	**66.0**	**73.9**	**48.6**	**66.5**	**1359**	**85.8**	**68.0**
FLLDM [52]	71.0	**90.8**	70.8	61.6	**71.4**	44.5	65.1	2018	**86.3**	66.1
TSA-LNet [3]	**74.1**	**92.7**	**72.9**	**63.5**	**75.4**	**46.1**	**66.5**	2013	**88.7**	**68.5**
Fast-CenLaneNet	**7** **2** **.** **7 ± 0.2**	89.4 ± 0.3	**7** **0.9 ± 0.2**	**6** **5** **.** **2 ± 1.3**	70.5 ± 1.0	**4** **7.9 ± 0.3**	**68.** **8 ± 1.0**	2866 ± 62	82.9 ± 0.3	**67.** **5 ± 0.2**

Note: The best, second, and third results are highlighted in bold black, bold red, and bold blue fonts, respectively. “-” indicates unavailable results. Except for Crossroad (FP↓), all results are reported as F-measure.

**Table 7:** Results on lane-changing scenarios.

Method	Normal	Crowded	Dazzle	Shadow	Night	Curve	Arrow	Total
Enet-SAD [22]	86.9 *	67.9 *	64.7 *	45.8 *	68.5 *	55.0 *	82.1 *	76.6 *
UltraLD [12]	85.5 *	64.8 *	71.8 *	72.3 *	68.8 *	56.4 *	82.3 *	76.5 *
Fast-CenLaneNet	**88.0 ± 0.8**	**69.9 ± 1.6**	68.7 ± 4.2	71.2 ± 5.9	**71.0 ± 2.1**	**68.5 ± 3.9**	76.3 ± 1.9	**77.6 ± 0.9**

Note: The best result is highlighted in bold black font. “*” indicates the corresponding values re-measured by us.

**Table 8:** Comparison of different attention mechanisms on TuSimple.

Attention	Acc (%)	FPR	FNR
PAM	96.29 ± 0.06	0.041 ± 0.002	0.033 ± 0.001
CBAM	96.29 ± 0.09	0.042 ± 0.002	0.032 ± 0.001
MHSA	96.29 ± 0.13	**0.037** ± 0.002	0.033 ± 0.002
LSSA	**96.40 ± 0.06**	0.043 ± 0.003	**0.029** ± 0.001

Note: The best result is highlighted in bold black font.

**Table 9:** Effect of the FC mapping structure in LSSA.

FC Mapping	Acc (%)	FPR	FNR	FPS
N-N	96.37 ± 0.05	0.044 ± 0.006	0.031 ± 0.002	165.9 ± 8.0
N-N/2-N (Ours)	**96.40 ± 0.06**	**0.043 ± 0.003**	**0.029 ± 0.001**	162.7 ± 6.8
N-N-N/2-N	96.36 ± 0.04	0.044 ± 0.002	0.030 ± 0.002	160.8 ± 4.0
N-N-N-N/2-N/2-N	96.33 ± 0.06	0.044 ± 0.005	0.031 ± 0.002	**160.0 ± 1.3**

Note: The best result is highlighted in bold black font.

**Table 10:** Effect of m in LSSA.

m	Acc (%)	FPR	FNR
0.25	96.38 ± 0.64	0.044 ± 0.003	0.030 ± 0.003
0.5 (Ours)	**96.40 ± 0.06**	**0.043 ± 0.003**	**0.029 ± 0.001**
0.75	96.35 ± 0.04	0.046 ± 0.003	0.031 ± 0.001
1	96.35 ± 0.04	0.045 ± 0.004	0.030 ± 0.001

Note: The best result is highlighted in bold black font.

**Table 11:** Performance comparison of different settings on TuSimple.

Backbone	LSSA	Acc (%)	FPS	Params (M)	FLOPs (G)
ResNet		96.13 ± 0.04	**189.9 ± 8.5**	0.9	9.9
ResNet	✓	96.19 ± 0.03	163.1 ± 3.9	5.1	12.1
LBN		96.34 ± 0.03	164.6 ± 3.1	**0.5**	**7.7**
LBN	✓	**96.40 ± 0.06**	162.7 ± 6.8	4.7	9.9

Note: The best result is highlighted in bold black font.

**Table 12:** Statistical reliability analysis of key ablation comparisons on TuSimple.

Comparison	n	ΔAcc (%)	95% CI of ΔAcc	*p*-Value
LBN vs. ResNet	5	+0.202	[0.123, 0.281]	0.002
LBN + LSSA vs. LBN	5	+0.060	[−0.019, 0.139]	0.103
ResNet + LSSA vs. ResNet	5	+0.052	[0.019, 0.085]	0.012
LBN + LSSA vs. ResNet + LSSA	5	+0.210	[0.144, 0.276]	<0.001

Note: Paired two-sided *t*-tests are conducted using repeated runs with the same random seeds. **Δ**Acc denotes the mean accuracy difference between the first and second configurations in each comparison.

## Data Availability

The benchmark datasets used in this work are openly available in TuSimple at http://github.com/TuSimple/tusimple-benchmark [48] (accessed on 10 July 2026) and in CULane at https://xingangpan.github.io/projects/CULane.html [16] (accessed on 10 July 2026). The code will be made publicly available at https://github.com/qidonghan/Fast-CenLaneNet on 30 July 2026.
